# The role of New World vultures as carriers of environmental antimicrobial resistance

**DOI:** 10.1186/s12866-024-03621-w

**Published:** 2024-11-20

**Authors:** Anaïs K. Tallon, Renotta K. Smith, Scott Rush, Adrian Naveda-Rodriguez, John P. Brooks

**Affiliations:** 1 Helmholtz Institute for Functional Marine Biodiversity at the University of Oldenburg (HiFMB), Ammerländer, Heerstrasse 231, 26121 Oldenburg, Germany; 2 USDA-ARS, Genetics and Sustainable Agriculture Unit, 150 Twelve Lane, Mississippi State, MS 39762-5367 USA; 3 Department of Wildlife, Fisheries, and Aquaculture, Mississippi State University, P.O. Box 9690, Mississippi State, MS 39762 USA

**Keywords:** Antibiotic resistance genes, Antimicrobial resistance, Enterococci, *Escherichia coli*, Landfills, Multidrug resistance, One Health, *Salmonella* spp., Season, Vultures

## Abstract

**Background:**

Although antibiotics have significantly improved human and animal health, their intensive use leads to the accumulation of antimicrobial resistance (AMR) in the environment. Moreover, certain waste management practices create the ideal conditions for AMR development while providing predictable resources for wildlife. Here, we investigated the role of landfills in the potentiation of New World vultures to disseminate environmental AMR. We collected 107 samples (soil, water, and feces) between 2023 and 2024, in different bird use sites (roosts, landfills and boneyards).

**Results:**

We isolated enterococci (EN), *Escherichia coli* (EC), and *Salmonella* spp*.* (SM), performed antibiotic susceptibility tests, and quantified the presence of antibiotic resistance genes (ARGs) within all samples. We identified EN, EC, and SM, in 50, 37, and 26 samples, from the three vulture use areas, respectively. AMR was mainly to aminoglycoside, cephalosporin, and tetracycline, and the prevalence of multidrug resistance (MDR) was 5.3% (EC), 78.2% (EN), and 17.6% (SM). Variations in bacterial abundance and AMR/MDR profiles were found based on the season, use site, and sample types, which was corroborated by ARG analyses.

**Conclusions:**

Our study suggests that landfills constitute a source of zoonotic pathogens and AMR for wildlife, due to readily available refuse input. Using non-invasive molecular methods, we highlight an often-ignored ecosystem within the One Health paradigm.

**Supplementary Information:**

The online version contains supplementary material available at 10.1186/s12866-024-03621-w.

## Introduction

Since the discovery of the first antibiotic (i.e., penicillin) by Alexander Fleming in 1928, the development and use of other classes of antimicrobials, which include substances that act against all microorganisms (bacteria, viruses, parasites, and fungi), have significantly decreased mortality and morbidity rates in humans and animals [1]. Yet, the overuse and misuse of antimicrobial drugs is tightly linked to the proliferation and spread of bacteria carrying antimicrobial resistance (AMR) properties in the environment, thereby perpetuating the global AMR crisis [2, 3]. As such, AMR is now one of the most significant threats to food security and the development of effective treatments against human and animal diseases. It is estimated that if no action is taken, AMR may cause 10 million annual deaths by 2050 worldwide, force 24 million people into extreme poverty and cause a reduction of almost 4% in livestock production globally, while jeopardizing the ability to treat common infectious diseases and undermining many other areas of medicine [2–4]. Because AMR is a global and complex concern of increasing magnitude that exists at the nexus of human, animal, and environmental health, AMR research must be considered in a One Health perspective.

In addition to the intensive use of antimicrobials, the surge of daily-renewed urban waste, as a result of growing human populations, consistently leads to profound changes in ecological systems. Although antimicrobial resistance genes (ARGs) and AMR bacteria are ubiquitously found in various types of samples, including soil, water bodies, wastewater, and even pristine environments with limited human impact, their prevalence was found to be exacerbated in environments exposed to human activities [5–7]. The accumulation of anthropogenically-sourced products in environments that have been substantially altered by people, such as landfills, not only creates ideal conditions for AMR development and transmission, but also provides substantial and predictable resources for wildlife [8]. Consequently, urban waste, through the increased exposure to AMR bacteria, was found to alter health and disease dynamics in a plethora of wildlife species, especially urban bird populations which coexist, and often exploit materials that are anthropogenically derived [9, 10]. Moreover, the combination of urban expansion, habitat loss and fragmentation of natural habitats ineluctably forces wildlife into closer contact with humans and domestic, facilitating zoonotic disease emergence and elevating transmission risks at the human-animal interface [10–12].

Although a positive correlation exists between spatial distance to anthropogenic wastes and the prevalence of ARGs, the role of “landfill ecosystems” to potentiate wildlife to amplify and disseminate AMR in the environment remains unclear. To date, most AMR research has been carried out in clinical settings, focusing on livestock and human health, and demonstrated that AMR prevalence in livestock and domestic animals is greatly influenced by increasing human activity and use of antibiotics [13–16]. Fewer studies have suggested that wildlife may also play a crucial role in spreading AMR, as data on the occurrence of AMR bacteria in wild animals, especially in areas with poor sanitation and inadequate waste management, remains largely overlooked [17–19]. Several authors have highlighted the importance of molecular and ecological investigations to understand the patterns, pathways, and mechanisms involved in AMR dissemination and maintenance involving wildlife populations [17, 18, 20]. Furthermore, because wild animals are highly mobile species, unlike livestock which represent relatively sessile bio-monitors, previous research suggested that even remote wildlife can acquire and spread AMR over long distances, most likely through dispersal [19, 21, 22].

In the context of landfills, anthropogenically-derived waste, and wildlife, we investigated whether New World vultures (*Coragyps atratus* and *Cathartes aura*) can function as bio-monitors in an approach to quantify AMR prevalence across specific spatial and temporal scales. The Black Vulture (*C. atratus*) and Turkey Vulture (*C. aura*) have a wide distribution across the Americas, although *C. aura* exhibits a broader range, extending north to southern Canada [23, 24]. These New World vultures inhabit a variety of habitats and ecosystems throughout the Americas, including deserts, tropical rainforests, coastal and mountain areas, but also inhabiting diverse natural and human-modified habitats [23]. The wide ranging of both *C. atratus* and *C. aura* implies that these species may cover large areas while foraging, potentially exposing them to diverse environmental conditions and pollutants. While *C. atratus* and *C. aura* have overlapping ranges, the home range sizes of both species vary throughout the year. Turkey Vultures keep consistent home ranges, except in May when space use decreases, likely due to chick-rearing, whereas Black Vultures have larger and more variable ranges, especially during the breeding season (January–April) [24, 25]. Due to their feeding habits (i.e., obligate scavenging), migratory behavior, and capacity to adapt to different environments, most vulture species, including *C. atratus* and *C. aura*, were previously described as potential reservoirs, sources, and amplifiers of AMR bacteria, as well as candidate sentinels for pathogen pollution in the environment [26–29].

In this study, we aimed to 1. Compare bacterial abundance of three different target microorganisms, 2. Identify AMR profiles, 3. Estimate the prevalence of multi-drug resistance (MDR), and 4. Characterize ARG expression profiles among 107 samples (feces, soil, and water) collected across various environments and a full-year cycle. As such, we were able to compare ‘natural’ background AMR (vulture roosts) and AMR recently acquired from anthropogenic sources (landfills). We targeted various microorganisms, including *Escherichia coli*, enterococci, and *Salmonella* spp., which are known as zoonotic fecal indicator bacteria and pathogens, respectively, and may further enhance the transfer of AMR in wildlife and environments [30–33]. These three key bacteria are present in the gastrointestinal flora of numerous animal species (including livestock and humans) and in a wide range of ecosystem components (i.e., soils, sediments, feces, freshwater, marine water, plants), and are opportunistic pathogens involved in human and animal infections [34–37]. Notably, *E. coli* was previously described as the most common microorganism identified in raptors with clinical signs of septicemia or respiratory disorders [37] and was the first bacteria where AMR was reported in wild birds [38]. In addition to AMR profiles, we also described abundance profiles of seven ARGs and the Integron-1 gene, which encodes for an integrase involved in the horizontal transfer of genes like those conferring AMR [39]. Because multi-drug resistance (MDR) was previously described in scavengers and considerably threatens the treatment of wild animals in rescue facilities and the implementation of conservation strategies [29, 40–42], we also decided to investigate the presence of MDR in our samples.

As landfills may act as stepping-stones in landscape connectivity between wild environments and human-made habitats for a plethora of species, this study provides valuable information regarding the distribution of AMR hotspots. A better understanding of the role of wildlife, particularly scavenging bird species, in AMR dissemination is key in addressing the future AMR crisis through the development of up-to-date surveillance and mitigation systems.

## Results

### Soil moisture

**Fig. 1:**
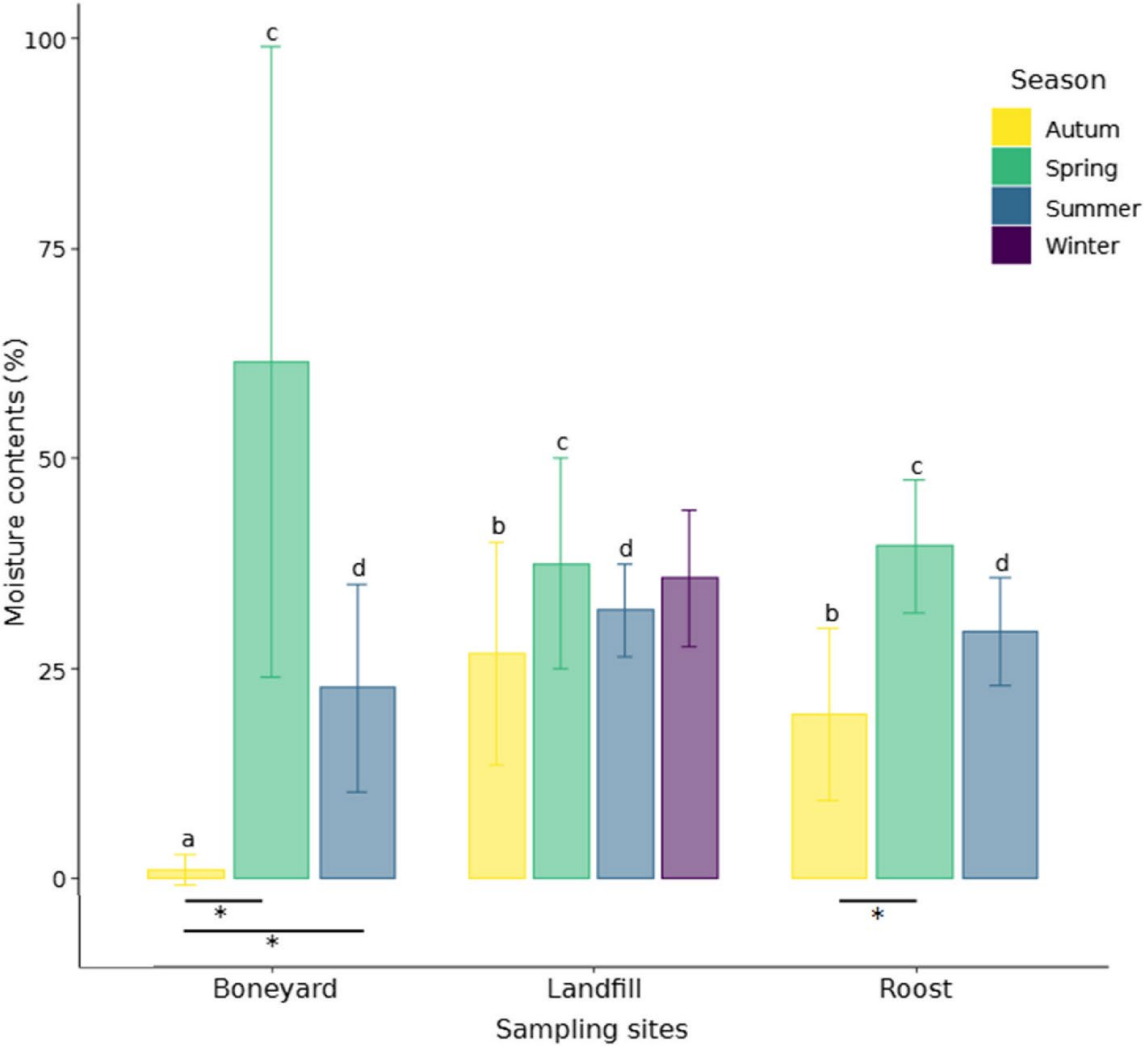
Moisture contents (%) in 10 g of soil cores collected at the different sampling sites (boneyards, landfills, and roosts), and across a full year-cycle (spring, summer, autumn, and winter). The error bars indicate standard deviations. The differences in soil moisture between seasons are indicated by the lower-case letters, and variations across sampling sites are indicated by the asterisk

Clear variations in the soil moisture were observed across seasons (df = 3, X^2^ = 21.22, *p*-value < 0.0001; Fig. 1). As such, samples collected at the boneyards contained more moisture in spring (61.54%; df = 62, H = 2.25, *p*-value = 0.023) than in autumn (3.06%). Similarly, samples collected at the roosts in spring showed higher moisture contents (39.51%; df = 62, H = 3.72, *p*-value = 0.0002) than samples collected during autumn (19.58%). No difference in soil moisture was found across seasons in the landfills. When comparing samples collected during autumn, boneyard samples (3.06%) contained significantly less moisture than in landfills (26.33%; df = 62, H = -2.04, *p*-value = 0.002) and the roosts (19.58%; df = 62, H = 1.36, *p*-value = 0.036).

### Bacterial abundance

Overall, *E. coli*, enterococci, and *Salmonella* spp. were isolated from a total of 50 (46.73%), 37 (34.58%), and 26 (24.3%) samples, respectively. The presence of these bacteria was confirmed through quantitative polymerase chain reaction (qPCR) in all isolates, except in one enterococci isolate. No *E. coli* or *Salmonella* spp. was found in any of the control soil samples, and no enterococci were identified in the control samples collected at the roosts (MER, STK1, STK2, and OR) and the boneyard PC (Table S3). While enterococci were isolated from some control samples collected at the roost GTR and both landfill sites (GTR and MER), the bacterial abundance was significantly lower than in the non-control samples (df = 1, F = 11.34, *p*-value = 0.029).

We did not find a statistically significant difference among seasons relative to bacterial abundance patterns. However, variations in the number of colony-forming units (CFUs) of both *E. coli* and enterococci were found across the different types of samples (*E. coli:* df = 2, F = 4.22, *p*-value = 0.017; enterococci: df = 2, F = 5.25, *p*-value = 0.007). In samples containing *E. coli,* higher proportions of the bacteria were found in fecal samples than from soil samples (t = 2.85, *p*-value = 0.014), with 96.73% of all successful isolations made from feces, 2.75% from soil, and 0.52% from water (Table 1). Similarly, more enterococci were found in feces, when compared to soil samples (t = 3.16, *p*-value = 0.002), with 17 successful isolations from fecal samples (91.63%), in soil (8.52%), and in water (0.12%; Table 1). While no statistically significant differences in abundance patterns of *E. coli* were found across the different sampling locations, we highlighted an interaction effect between the type of sample and the sampling location on enterococci abundance patterns (df = 3, F = 6.3, *p*-value = 0.001). As such, in landfills, more bacteria were isolated in feces than in soil (t = 5.33, *p*-value < 0.0001) and water samples (t = 4.57, *p*-value < 0.0001; Table 1). Overall, we found a positive correlation between the abundance of *E. coli* and enterococci (tau = 0.56; *p*-value < 0.0001).

**Table 1:** Total bacterial abundance of *E. coli* and enterococci in soil, fecal, and water samples collected in boneyards, landfills, and roosts. As no seasonal effects were found, the colony forming unit (CFU) was summed over all season for both *E. coli* and enterococci

Sampling Site	Type of Sample	Total CFU from Successful Isolation	
		***E. coli***	**Enterococci**
Roost	Feces	150,200	110,100
Roost	Soil	310,070	420
Roost	Water	0	0
Landfill	Feces	13,360,740	9,230,400
Landfill	Soil	68,390	264,370
Landfill	Water	3,930	13,160
Boneyard	Feces	7,538,800	796,300
Boneyard	Soil	220,090	680,130
Boneyard	Water	110,080	0

Among *Salmonella* spp. isolates, we found variations in bacterial successful isolations based on the sampling location (df = 2, F = 7.04, *p*-value = 0.001), with 56.2% of successful isolations made in the samples collected at the boneyards (Table 2). Variations in bacterial presence/absence were also found across the season (df = 3, F = 4.74, *p*-value = 0.004), with less *Salmonella* detected in spring samples (10.24%) than in winter samples (41.98%; Table 2). No changes in the frequency of detection of *Salmonella* were found between sample type, however, we showed an interaction effect between the type of sample and the sampling location, with significantly more *Salmonella* isolated in fecal samples collected at the boneyard (83.33%), when compared to the landfills (df = 98, t = 4.24, *p*-value < 0.0001), and the roosts (df = 98, t = 3.2, *p*-value = 0.005; Table 2).

**Table 2:** The rate of *Salmonella* spp. isolation is reported in each isolate through presence/absence. The proportion of positive samples is represented by sampling site, season, and type of sample, and shows the number of positive samples/total number of tested samples for each condition

Condition	Proportion of Positive Samples	
*Sampling Site*		
Boneyard	15/32	56.2%
Landfill	15/52	34.58%
Roost	2/26	9.22%
*Season*		
Spring	6/41	10.24%
Summer	10/27	25.91%
Autumn	10/32	21.87%
Winter	6/10	41.98%
*Type of Sample—Feces*		
Boneyard	10/12	83.33%
Landfill	0/6	0%
Roost	1/6	16.67%

### Antimicrobial resistance profiles

#### Prevalence of AMR

**Fig. 2:**
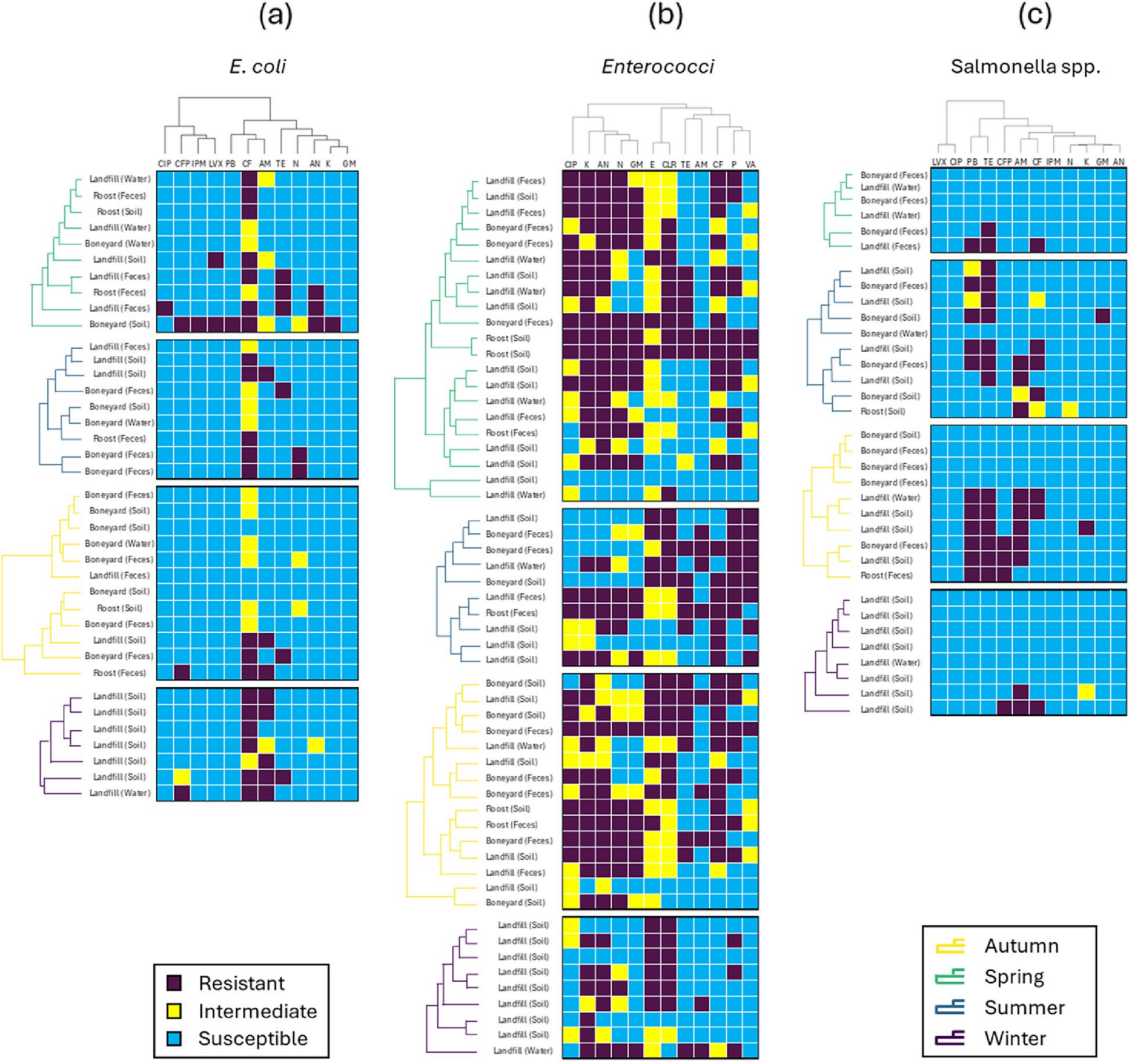
Heatmaps showing the antimicrobial resistance of (**a**) *E. coli*, (**b**) enterococci, and (**c**) *Salmonella* spp. in soil, fecal, and water samples collected in boneyards, landfills, and roosts. Dendrograms present the hierarchical cluster analysis of the samples (rows) clustered by seasons, and the following antibiotics (columns): erythromycin (E), neomycin (N), kanamycin (K), gentamicin (GM), cephalothin (CF), penicillin (P), ciprofloxacin (CIP), tetracycline (TE), vancomycin (VA), ampicillin (AM), clarithromycin (CLR), Amikacin (AN), cefoperazone (CFP), ciprofloxacin (CIP), levofloxacin (LVX), and polymyxin-B (PB). Antibiotic susceptibility is indicated by color (purple: resistant, yellow: intermediate, blue: susceptible)

Resistance profiles of *E. coli* were influenced by the season (df = 3, X^2^ = 10.32, *p*-value = 0.016) and were significantly different across antibiotics tested (df = 11, X^2^ = 198.08, *p*-value < 0.0001). However, the prevalence of AMR did not vary significantly between boneyards, roosts, and landfills, and was similar across the different sample types. Isolates from summer and autumn showed similar AMR profiles across antibiotics with less samples showing antibiotic resistance (Fig. 2a). In contrast, isolates from spring and winter exhibited higher antibiotic resistance in both the number of antibiotics and the proportion of resistant samples (Fig. 2a). Noteworthy, all the samples collected in winter (which were all from landfills) showed a resistance to at least one antibiotic. The highest resistance of *E. coli* isolates was seen against the following antibiotics: CF (55.26 %) and AM (21.05%), and all samples were found to be susceptible to GM (Fig. 2a).

In contrast to *E. coli*, resistance profiles of enterococci isolates were influenced by the sampling location (df = 2, X^2^ = 37.38, *p*-value < 0.001), as samples collected in landfills displayed a resistance to 4.42 antibiotics on average, while samples collected in boneyards and roosts were resistant to 6.38 and 8.5 antibiotics, respectively (Fig. 2b). Antibiotic resistance was also influenced by the type of sample (df = 2, X^2^ = 20.12, *p*-value < 0.001). Higher resistance was found in fecal samples in landfills (H = -2.87, p-value = 0.004), when compared to soil samples, while a higher resistance was described in soil samples collected in roosts (H = 2.16, *p*-value = 0.031; Fig. 2b). In addition, we found less resistance in the isolates collected in winter than during the other seasons (df = 3, X^2^ = 20.76, *p*-value < 0.001), when comparing all soil samples collected at landfills (Fig. 2b). Similar to *E. coli*, the type of antibiotics also influenced the AMR profiles of enterococci (df = 11, X^2^ = 92.09, *p*-value < 0.001). All samples combined, the highest resistance of enterococci isolates was seen against the following antibiotics: K (70.91%) and N (69.09%), while the lowest resistance was against AM (20 %) and VA (20%) (Fig. 2b). None of the control samples where enterococci was isolated (i.e., the roost GTR and the landfill sites GTR and MER) showed AMR.

Prevalence of AMR in *Salmonella* isolates was also influenced by the season (df = 3, X^2^ = 16.78, *p*-value < 0.001), and the type of antibiotics (df = 11, X^2^ = 89.5, *p*-value < 0.001). In contrast with *E. coli*, a higher proportion of summer and autumn samples presented AMR, when compared to spring and winter samples (Fig. 2c). For instance, landfill samples collected during autumn were all resistant to at least 4 antibiotics, which was more than samples collected in spring (H = -2.31, *p*-value = 0.021), summer (H = -1.79, *p*-value = 0.074), and winter (H = -4.44, *p*-value < 0.001). Noteworthy, during autumn, samples collected in landfills showed a higher resistance than isolates from boneyards (H = 3.48, *p*-value < 0.001). The highest resistance of *Salmonella* isolates was seen against TE (44.12%) and PB (32.35%) (Fig. 2c). The following antibiotics: LVX, CIP, IPM, N, and AN, did not induce any resistance in any of the isolates (Fig. 2c). The prevalence of AMR in *Salmonella* isolates did not vary between the different sample types.

#### Prevalence of multidrug resistance

**Fig. 3:**
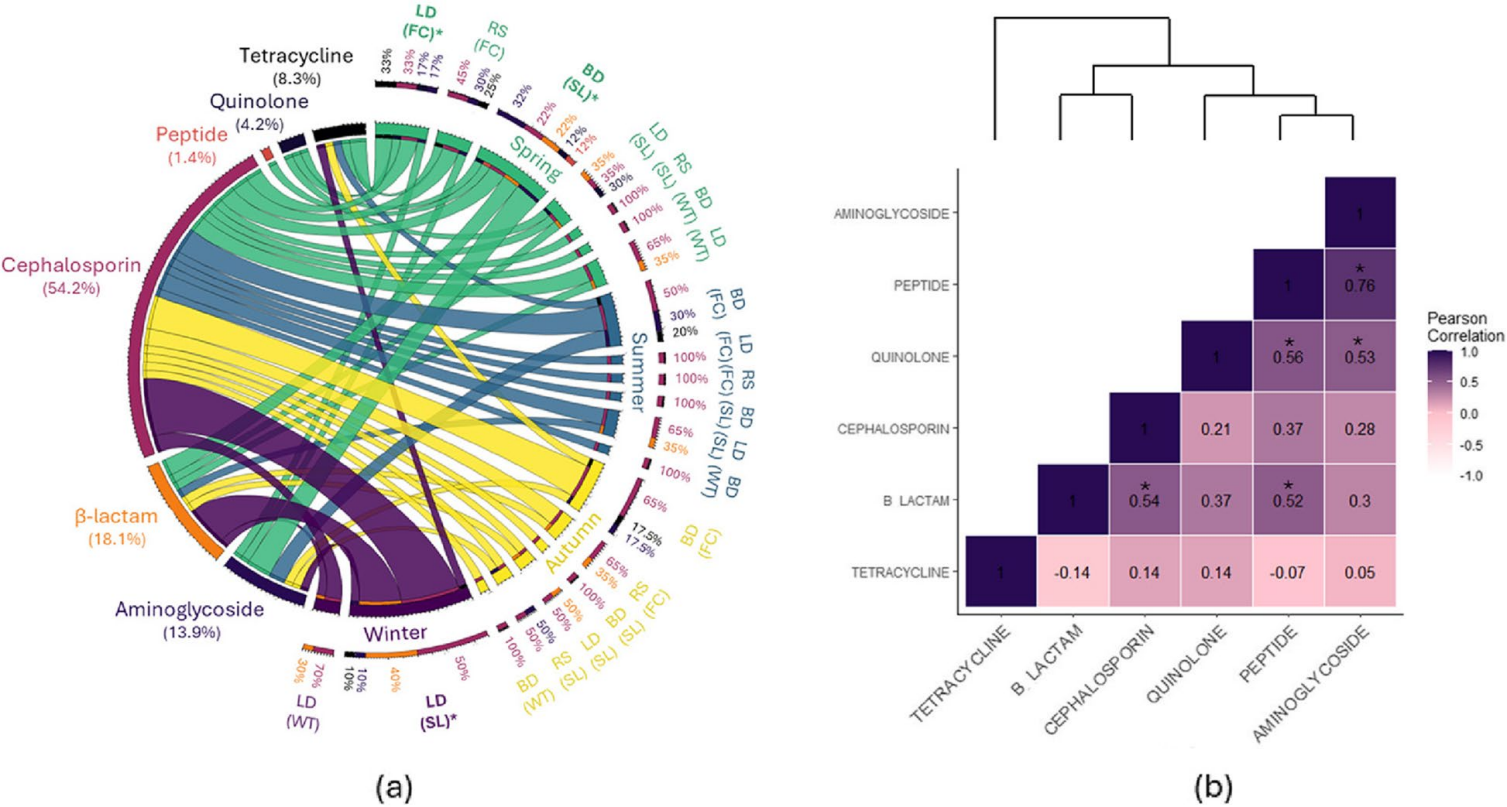
**a** The prevalence of multidrug resistance in *E. coli* isolates, based on the sampling locations (BD: boneyard, LD: landfill, and RS: roost) and the type of samples (FC: feces, SL: soil, and WT: water), is shown for each season (Spring: green, Summer: blue, Autumn: yellow, and Winter: purple). The inner circles and the width of the ribbon of the circles represent the isolates (right) and show the proportion of resistance to each of the following antimicrobial classes (left): aminoglycoside, β-lactam, cephalosporin, glycopeptides, quinolone, and tetracycline. Samples with multidrug resistance (> 3 antimicrobial classes) are in bold letters and highlighted by an asterisk. Proportions < 10% are shown by **. **b** The dendrogram and heatmap are based on the Pearson correlation coefficient and show the main association between antimicrobial classes. A negative value indicates a negative linear correlation, while 0 means no correlation, and positive values indicate a positive correlation

**Fig. 4:**
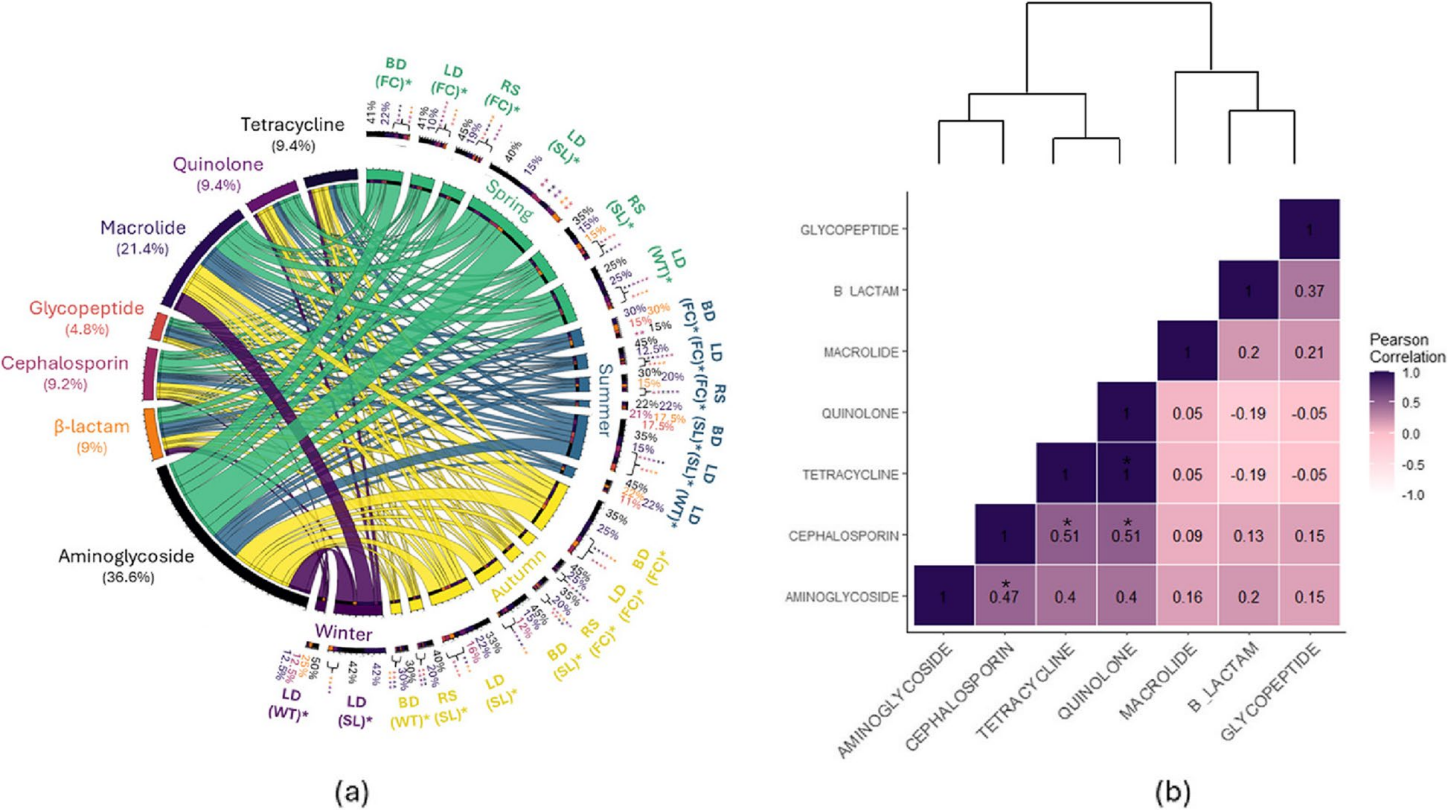
**a** The prevalence of multidrug resistance in enterococci isolates, based on the sampling locations (BD: boneyard, LD: landfill, and RS: roost) and the type of samples (FC: feces, SL: soil, and WT: water), is shown for each season (Spring: green, Summer: blue, Autumn: yellow, and Winter: purple). The inner circles and the width of the ribbon of the Circos diagram represent the isolates (right) and show the proportion of resistance to each of the following antimicrobial classes (left): aminoglycoside, β-lactam, cephalosporin, glycopeptide, macrolide, quinolone, and tetracycline. Samples with multidrug resistance (> 3 antimicrobial classes) are in bold letters and highlighted by an asterisk. Proportions < 10% are shown by **. **b** The dendrogram and heatmap are based on the Pearson correlation coefficient and show the main association between antimicrobial classes. A negative value indicates a negative linear correlation, while 0 means no correlation, and positive values indicate a positive correlation

**Fig. 5:**
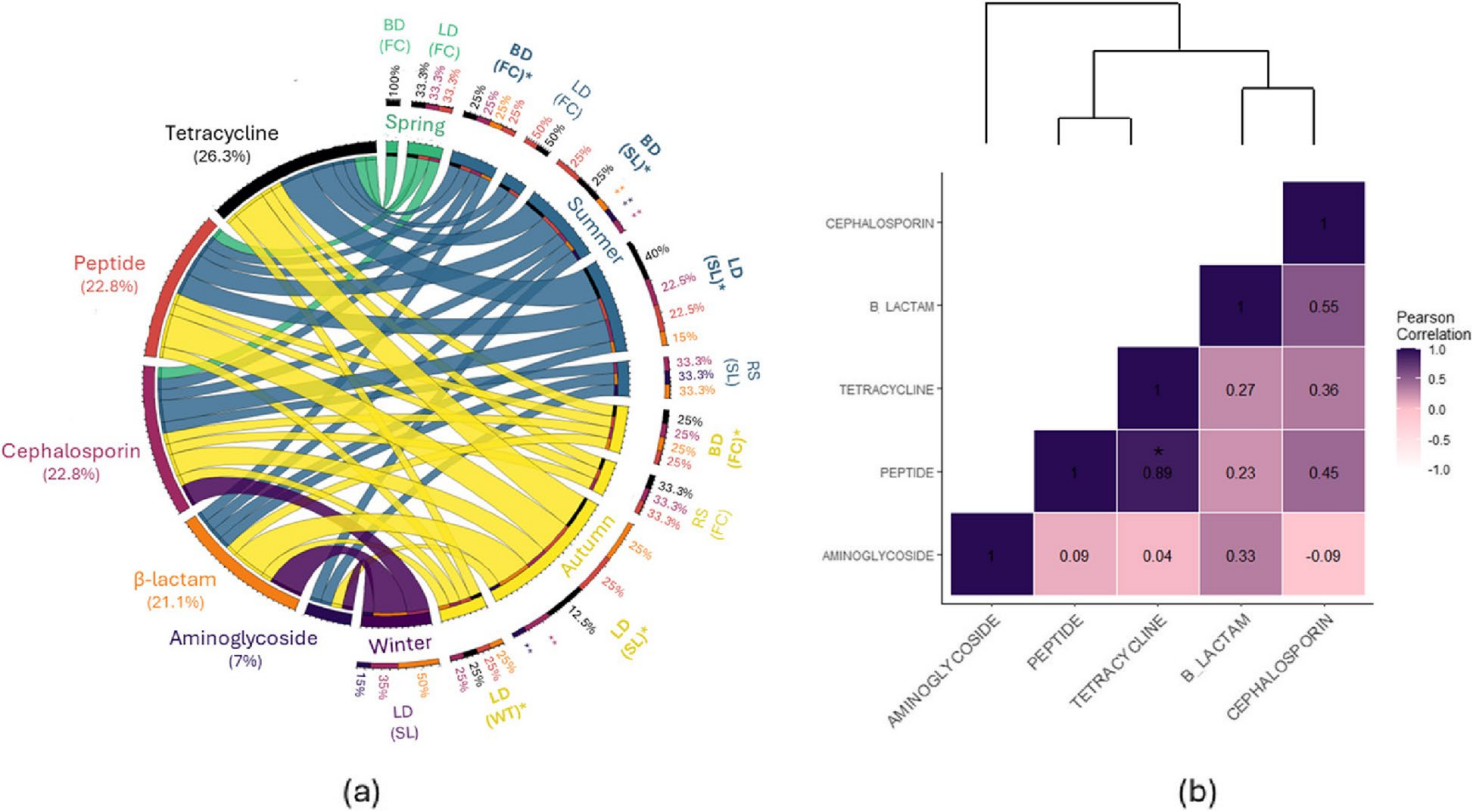
**a** The prevalence of multidrug resistance in *Salmonella* isolates, based on the sampling locations (BD: boneyard, LD: landfill, and RS: roost) and the type of samples (FC: feces, SL: soil, and WT: water), is shown for each season (Spring: green, Summer: blue, Autumn: yellow, and Winter: purple). The inner circles and the width of the ribbon of the Circos diagram represent the isolates (right) and show the proportion of resistance to each of the following antimicrobial classes (left): aminoglycoside, β-lactam, cephalosporin, peptide, and tetracycline. Samples with multidrug resistance (> 3 antimicrobial classes) are in bold letters and highlighted by an asterisk. Proportions < 10% are shown by **. **b** The dendrogram and heatmap are based on the Pearson correlation coefficient and show the main association between antimicrobial classes. A negative value indicates a negative linear correlation, while 0 means no correlation, and positive values indicate a positive correlation

From all samples combined, we found that AMR in *E. coli* isolates was mainly to β-lactam (18.06%) and cephalosporin (54.17%; Fig. 3a). Most enterococci isolates exhibited AMR to aminoglycoside (36.64%) and macrolide (21.43%; Fig. 4a), while a prevalence of resistance to glycopeptides (22.81%), tetracycline (26.32%), and cephalosporin (22.81%) was found among *Salmonella* isolates (Fig. 5a). The prevalence of MDR (>3 antibiotic classes) in *E. coli*, enterococci, and *Salmonella* isolates was 5.3%, 78.2%, 17.6%, respectively. enterococci isolates exhibited MDR across the four seasons, with the largest MDR in soil (landfill) and fecal (boneyard) samples (Fig. 4a). In contrast, only *E. coli* samples collected in spring and winter showed MDR (Fig. 3a), while only *Salmonella* samples collected in summer and autumn showed MDR (Fig. 5a). In line with enterococci, only *E. coli* and *Salmonella* samples that were collected in landfills and boneyards exhibited MDR, with the largest MDR among soil samples (Figs. 3a, 4 and 5a).

We found significant positive correlations between resistance of *E. coli* to aminoglycoside and peptide (*r* = 0.76, *p*-value = 0.001) and quinolone (*r* = 0.53, *p*-value = 0.042), as well as between peptide and quinolone (*r* = 0.56, *p*-value = 0.03) and β-lactam (*r* = 0.52, *p*-value = 0.046), and between cephalosporin and β-lactam (*r* = 0.54, *p*-value = 0.04; Fig. 3b). In contrast, positive association were found in enterococci isolates between resistance to quinolone and tetracycline (*r* = 1, *p*-value < 0.001) and cephalosporin (*r* = 0.51, *p*-value = 0.022), as well as between resistance to cephalosporin and tetracycline (*r* = 0.51, *p*-value = 0.022) and aminoglycoside (*r* = 0.47, *p*-value = 0.04; Fig. 4b). Among *Salmonella* isolates, a positive correlation was only described between resistance to tetracycline and peptide (*r* = 0.89, *p*-value = 0.001; Fig. 5b). We found no negative correlations among the MDR profiles.

### ARG expression profiles

Of all the collected samples, 86 soil and fecal samples were tested for the presence of ARGs. The 16S rRNA was successfully identified in all samples, while the class 1 integron was found in 72 (83.7%) samples. The β-lactam resistance gene *bla*TEM and the sulfonamide resistance gene *sul*1 were identified in 74 and 76 samples, respectively. Overall, the macrolide resistance genes *erm*A and *erm*F were the most found ARGs with 76 (90.5%) and 79 (91.9%) samples carrying these genes, respectively. In contrast, tetracycline resistance was identified in only 64 (76.2%; *tet*A) and 48 (55.8%; *tet*B) samples.

**Fig. 6:**
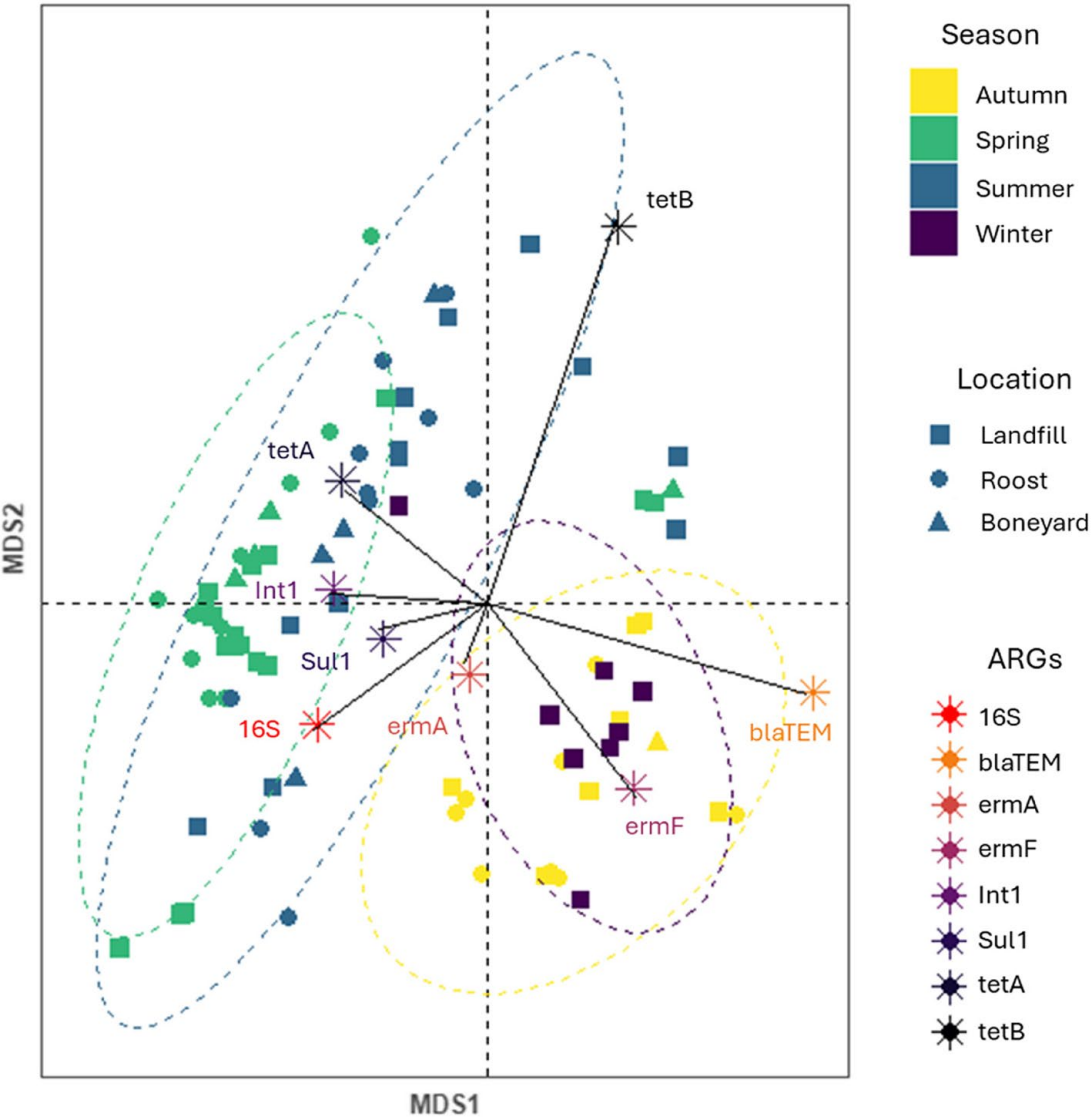
Nonmetric multidimensional scaling (NMDS) plot ordination of Bray–Curtis (stress = 0.07) showing the antimicrobial resistance gene analysis. Each sample is represented by a point, whose shape encodes for the type of sampling type (circle: roosts, square: landfill, triangle: boneyard), and the color encodes for the season (yellow: Fall, green: Spring, blue: Summer, yellow: Autumn, and purple: Winter). The ellipses represent the standard deviation around the centroid of each respective season cluster. Vectors (arrows) represent each target ARG with a length proportional to their importance as an explanatory environmental variable. ARG profiles are significantly different between seasons (ANOSIM, R = 0.334, *p*-value < 0.001)

The ANOSIM revealed that the presence of ARGs identified during qPCR was not influenced by the sampling locations (*R* = -0.04, *p*-value = 0.853), nor the sample type (*R* = 0.068, *p*-value = 0.203). However, the season had a significant influence on the observed variations in ARG profiles (*R* = 0.334, *p*-value < 0.0001). The NMDS showed a separation between samples collected during autumn and winter from samples collected in spring and summer (Fig. 6). The ISA revealed that *sul1* was statistically more abundant in samples collected in autumn (stat = 0.375, *p*-value = 0.017), while *tet*A was identified as an indicator of winter samples (stat = 0.374, *p*-value = 0.021). In addition, the ARGs *erm*F (stat = 0.645, *p*-value < 0.001) and *bla*TEM (stat = 0.365, *p*-value = 0.02), and the 16S rRNA (stat = 0.508, *p*-value = 0.001) were more associated with the group of autumn and winter samples.

## Discussion

The present study investigated the role of New World vultures as potent AMR reservoirs, to understand the presence of environmental AMR in landfills and wildlife through soil, water, and feces. These birds were chosen for their scavenging habits and because they are found living in close proximity to humans and can readily feed on urban waste. Although we cannot demonstrate a direct transmission of AMR between the landfills and the vultures, we found that both the bacterial abundance profiles and the prevalence of AMR/MDR were influenced by the seasons, sample type, and sampling locations. Moreover, our study revealed the presence of ARGs associated with aminoglycosides and sulfonamides as the most found gene families.

In line with previous work on wild birds, including vultures, we found that the prevalence of *E. coli* and enterococci was higher than *Salmonella* spp. [40–42], and showed that the levels of AMR in bacteria among bird populations are not static [43]. The highest occurrence of *Salmonella* was in spring, which coincides with the peak of vulture breeding season in the southern U.S., while *Salmonella* isolates exhibited the highest AMR in summer and autumn (post-breeding periods). Our results concurred with previous research showing that the seasonal cycle of *Salmonella* largely overlaps with the breeding season in wild birds [27, 41]. Although we did not find any seasonal impact on bacterial abundance of *E. coli* and enterococci*,* isolates collected during spring (i.e., season with the highest moisture levels) exhibited higher AMR and MDR in *E. coli.* In contrast, high moisture levels coincided with lower AMR-enterococci, which aligns with previous findings where the prevalence of MDR-enterococci in environmental samples was lower during the rainy season compared to the dry season [44]. Our study suggests that seasonal changes and variations in soil moisture do not necessarily impact bacterial profiles but can influence AMR prevalence. Due to our uneven number of samples collected across seasons and the limited literature available on seasonal patterns of AMR prevalence, we suggest further investigation to characterize the role of seasonal fluctuations in AMR carriage by wildlife hosts.

Among *Salmonella* isolates, we found a difference between ‘natural’ background AMR (roosts and boneyards) and the AMR recently acquired from anthropogenic sources. While samples collected at the large roosts (“boneyards”) harbored more *Salmonella*, landfills presented higher AMR burden than samples collected in the natural sites, suggesting that bacterial abundance is not necessarily linked to the AMR profiles. The high abundance of *Salmonella* in these large roosts, where many bird carcasses were found, concurs with the environmental stability of *Salmonella* spp. to accumulate and persist in soil for several months, given specific environmental conditions [45, 47]. Unlike previously hypothesized, the present study emphasizes that the ability of wild birds in spreading *Salmonella* is likely not limited to areas that are highly contaminated by human waste (e.g., landfills) or domestic animal manure [46], but can also be found in roosts.

In contrast to *Salmonella* isolates, we found that bacterial abundance of *E. coli* was similar between the roosts, boneyards, and the landfills. Similarly, we could not distinguish between ‘natural’ background AMR from AMR recently acquired from anthropogenic sources (landfills) in *E. coli* isolates. The high carriage rates of *E. coli* and AMR in both the environment and bird feces, independently of the level of anthropogenic pressures, may be a result of the common transmission of this bacteria among wild birds *via* feces and water [29], and the relatively close proximity of all our sampling sites to urban settings. For instance, the boneyards were located less than 5 km from the closest human settlement, and birds living in the large roost GTR were seen feeding in the landfills. Similarly, the roost sites STK1 and STK2 were located less than 10 km from human settlements, and OR was found less than 1 km from the town of Macon, MS (approximately 3,000 inhabitants). Such distances are relatively short in comparison to previous work highlighting differences in the prevalence of resistant *E. coli* between gull populations from Middleton island and mainland Alaska (> 100 km) [43].

More surprisingly, while the abundance of enterococci was similar between the natural sites and landfills, we found a higher prevalence of ‘natural’ background AMR (roosts and boneyards) than AMR recently acquired from anthropogenic sources. Our results contrast with the assumption that AMR would be higher in birds living in urban and landfill settings, which are more likely to be impacted by anthropogenic activities (e.g., untreated wastewater, human and medical waste), therefore, increasing the risk of AMR introduction into the environment [43, 47]. Although there is still limited direct evidence comparing AMR and bacterial prevalence between natural and urban settings, we hypothesize that high prevalence of resistant enterococci in natural habitats may be linked to the accumulation of bird carcasses, as we suggested for *Salmonella*.

The high prevalence of resistant enterococci may also be explained by specific plasticity of *Enterococcus* spp. genome, which allows the bacteria to acquire AMR genes from gut bacteria and/or the environment more efficiently than *E. coli* [48]. Moreover, as shown for *Salmonella* spp., enterococci can persist in the environment for extended durations [49, 50]. For instance, the maintenance of AMR in vulture nesting areas was hypothesized to be linked to the presence of MDR bacteria in the food brought by the adults to the chicks (e.g., carcasses leftovers from landfills) [40]. However, because we did not have any information regarding the food sources or the presence of potential supplementary feeding stations used by the birds from the roosts and the boneyard PC in our study, we cannot infer whether the high prevalence of resistant enterococci is directly linked to the birds’ diet, like previously shown in avian scavengers in Europe [27].

In this study, we should note that the prevalence of AMR also depended on the sample type. Overall, we found higher bacterial abundance and AMR prevalence in fecal samples than in soil and water (*E. coli,* enterococci and *Salmonella* spp.), as demonstrated in European wild birds [40, 42, 46]. Yet, we acknowledge the heterogeneity in the availability of sample types, as 25% of the samples collected in the roosts and boneyards were feces (against only 7% of the samples collected at the landfills). This may have contributed to the higher AMR levels observed in the natural sites. Nevertheless, as our results reinforce the theory that wildlife can shed resistant bacteria into the environment through their feces [46, 51, 63], we suggest that the maintenance of AMR in vulture nesting areas may be linked to the potential contact in the nest between the chicks and adult feces, as previously advocated [40].

In line with previous studies showing a positive correlation between the prevalence of AMR carried by wildlife and the distance to urban environments and population size [43, 53], we suggest that the high prevalence of resistant enterococci in the natural sites may also be a reflection of their distance to poultry facilities and aquatic settings. On average, the roosts and boneyards were located 8.3 km from the closest poultry facility (against 14.8 km for the landfills), which concurs with previous work showing that AMR bacteria and genes originating from livestock carcasses can accumulate in animal production environments [52–54]. Similarly, rapid dissemination of AMR mediated in wild birds through horizontal transfer between farmlands and poultry environments has been widely documented [55–57]. Moreover, the natural sites of our study were also located less than 1 km from the closest aqueous settings (against 12.8 km for the landfills), which aligns with previous research showing that aquatic environments (e.g., rivers, lakes, and coastal areas), can serve as major transmission vectors for AMR pathogens, such as *E. coli* and *Salmonella* spp. [58–61].

Lastly, the high prevalence of resistant enterococci in the roosts may also result from the close proximity with human environments (e.g., gardens, roads, and wastewater treatment), as enterococcus spp. originating from human feces have greater persistence in environmental and aqueous environments than *E. coli* [62]. Yet, our results should be treated cautiously as the correlations between the distance to the sampling sites and human activities and/or aquatic settings do not take in account the distance and directions of the bird movements, nor the amount of poultry or the size of aquatic settings. As such, further investigation remains needed to confirm whether the high prevalence of AMR in natural habitats is linked to the likelihood of human isolates colonizing wild birds.

The present study highlights the omnipresence of CF resistance in *E. coli* isolates, regardless of the sample type, which concurs with other findings in both environmental and wildlife samples [31, 36, 40]. However, the high frequency of resistance phenotypes to cephalothin (54.17%) was higher than previously reported in birds [31, 40, 43, 46] and mammals [31, 46]. The highest resistance of *Salmonella* isolates was seen against TE (44.12%), which is one of the most commonly reported antimicrobial resistance phenotypes in various wildlife sources, including vultures [40], as well as mammals, water, and soil samples [63]. These findings are not surprising since cephalothin and tetracycline are older drugs widely used in therapy and livestock production systems since the 1940’s in the United States [27]. Besides, both of these drugs have shown increasing resistance trends in the past decades in animals, even though decreasing resistance trends were observed in humans [36]. However, unlike previous findings in wild and urban birds [13, 28, 40, 43], and other animal sources [36, 46], we found relatively little resistance to TE (15.8%) among our *E. coli* isolates. This low frequency of resistance phenotypes to TE coincides with the low prevalence of genes conferring resistance to tetracyclines (i.e., *tet*A and *tet*B) observed in our samples. Even though high prevalence of *tet* genes was shown in opportunistic bird species (e.g., crows and pigeons), our results are in accordance with the hypothesis that free-living birds harbor lower frequency of resistance to TE in *E. coli*, when compared to poultry environments [52, 64]. It should be noted that the U.S. Center for Disease Control and Prevention (CDC) doesn’t have CF and TE resistance as serious or concerning AMR threats according to their recent threat assessment [65]. However, the presence of resistant *Salmonella* spp. is listed as “serious” within this threat assessment.

We found a relatively high prevalence of resistance phenotypes to AM in *E. coli* (21.05 %), as previously shown in wild birds [36, 52]. Yet, the proportion of AM-resistant described in our study remains lower than previously shown (>65%) in gulls, white storks [13], buzzards [65], and vultures [28, 40] in Europe, which may be explained by different legislations and approval timelines between Europe and North America [52]. Moreover, phenotypic resistance of AM-resistant *E. coli* was shown to be correlated with the presence of the *bla*TEM gene in buzzards in Portugal [65], and wild birds in Poland [66]. While higher resistance to AM is generally found in poultry facilities and in birds living under higher anthropogenic pressures [43], we found no influence of the type of sampling site on ARG profiles. However, our study revealed that the expression patterns of *bla*TEM correlated with the seasons as almost all *E. coli* samples collected in winter (>70%) showed resistance to AM. These genes are of particular interest as they may belong to the extended-spectrum beta-lactamase (ESBL) producing enterobactericae, which are still considered “serious” threats according to the CDC [65]. In contrast to the high prevalence of resistance phenotypes to AM in *E. coli,* the lowest resistance in enterococci isolates was against AM (20%).

Despite the wide use of aminoglycosides (e.g., GM) in the poultry industry, which resulted in high prevalence of GM-resistance in chickens since their introduction in the 1960’s [36], all our sample isolates were susceptible to GM, which is in line with previous research in wildlife [18, 40, 42, 43, 46, 67]. These results also reflect the considerable efforts made by the poultry industry to reduce their antibiotic usage at the broiler production stage (i.e., the majority of antibiotic use being now limited to hatcheries). Even though several studies demonstrated a very high percentage (75%) of GM-resistant *E. coli* in ducks, eagles [52], vultures and seagulls [52], it was hypothesized that this high resistance may be the result of the production of other aminoglycoside-modifying genes. In contrast to the absence of GM resistance our *E. coli* isolates, the highest prevalence of resistance phenotypes in enterococci was seen against other aminoglycosides: K (70.91%) and N (69.09%), which was higher than in buzzards [66] and other wildlife [66]. Despite limited information about the prevalence of K and N resistance in enterococci isolated from wildlife, it was previously suggested that resistance to high levels of aminoglycosides like GM is frequently acquired through ARGs, while resistance to low levels of aminoglycosides, such as K and N, is often caused by intrinsic factors [67, 68]. As such, further investigation regarding the high prevalences of aminoglycoside resistance described in our enterococci isolates through genotypic analyses is needed.

We showed a high prevalence of genes conferring resistance to sulfonamide in all our samples, which concurs with previous studies showing that resistance to sulfonamide was also one of the most common resistance in wild birds [13, 52]. In North America, resistance to sulfonamide has been observed in humans since the 1950’s, and sulfonamides are one of the most commonly used antimicrobials in animal production systems to prevent or treat parasitic diseases in poultry and is now frequent in bacteria from farm animals [36]. As sulfonamide resistance exhibits an increasing trend in animals, previous studies have shown that a high prevalence of sulfonamide-resistant *E. coli* and *Salmonella* is associated with acquisition of the ARGs *sul1*, *sul2*, and *sul3* [69, 70]. Although we showed that the presence of *sul1* was more abundant in samples collected in autumn, more research is needed to investigate the potential seasonality in the carriage and subsequent expression of these genes.

In line with previous findings advocating that differences in the prevalence of MDR *E. coli* are reflective of geographic isolation [43], the incidence of MDR in resistant bacteria phenotypes was different across our sampling sites. Only the samples collected in landfills and boneyards exhibited MDR, suggesting that such environments may act as MDR reservoirs. In our study, the prevalence of MDR in *E. coli* and *Salmonella* isolates was relatively lower than previously described in humans and food animals [36], as well as in wild birds [13, 29, 52]. In agreement with previous work, we also showed that vultures, like other wild birds, are common carriers of MDR fecal bacteria [65]. Like AMR profiles, MDR also depended on the season, as only *E. coli* samples collected in spring and winter (e.g. vulture breeding) showed MDR, while only *Salmonella* samples collected in summer and autumn (post-breeding) showed MDR. The most common MDR profile was peptide-quinolone in resistant *E. coli* isolates, tetracycline-peptide in *Salmonella*, and cephalosporin-tetracycline in enterococci isolates. While these findings are in line with previous research on wildlife [13, 36, 40], our results highlight the complexity of MDR profiles in vultures, based on the sample type, anthropogenic pressure, and seasons. In the present study, the presence of potentially virulent *Salmonella* spp. together with a high prevalence of MDR phenotypes in vulture populations is particularly concerning and highlights the challenges in developing up-to-date surveillance and mitigation systems in the context of One Health.

## Materials and methods

### Study area and period

The study was carried out in East-Central Mississippi, USA at three different types of location; three vulture roosting sites, the recreation site Pratt Camp located in Aliceville Lake, near Columbus, and two landfills located in Oktibbeha and Lauderdale counties. The roosting sites (STK1: 33° 21' 13.3956"N, -88° 47' 38.832"W; STK2: 33°18'48.8802"N, -88°47'51.7986"W; and OR: 33°5'35.7324"N, -88°34'20.5278"W) corresponded to an old barn in a private garden, an abandoned forest house, and a dilapidated building from an old brick factory, respectively. Both STK1 and STK2 were in a radius of less than 10 km from human settlements (e.g., Starkville town center) and natural areas (e.g., Oktibbeha County Lake and Tombigbee National Forest). The roost OR was located along the Noxubee River, less than 1 km from the town Macon, and 10 km from a poultry facility.

Pratt Camp (PC; 33°20'48.1776"N, -88°24'9.936"W) was surrounded by Moores Bluff and the Tennessee-Tombigbee Waterway. Consequently, the sampling sites were only accessible by boat. The site PC was less than 5 km from the closest human settlement, 10 km from the Mississippi-Alabama border, and approximately 12 km from poultry farms. A considerable number (> 50) of bird carcasses was reported at this site during the spring and summer sample collection (while the timeline corresponds with bird avian influenza outbreaks in the region, we do not have any data to confirm the exact cause of these deaths).

The landfills were the Golden Triangle Regional (GTR) Solid Waste Management Authority (33°31'42.8874"N, -88°40'20.3268"W) and the Meridian (MER) Pine Ridge Sanitary Landfill (32°28'59.0448"N, -88°38'13.7934"W). Both sites shared geographical proximity with natural and aquatic areas; GTR was located 17.5 km flying distance from Columbus Lake, 27.5 km from Bluff Lake and Noxubee National Wildlife Refuge and 39.3 km from Tombigbee National Forest, while MER was 6.3 km from Long Creek reservoir and 20.8 km from Okatibbee Lake and state wildlife area. These landfills were relatively close to human settlements as GTR was located 14.6 km from Starkville, 9.6 km from West Point town centers, and 11.7 km from Golden Triangle Regional airport, whereas MER was 8.8 km from Meridian town center and 13.4 km from Meridian Regional Airport. At least 8 poultry facilities were found in a radius of less than 30 km around GTR, and a poultry farm was located less than 1 km from MER.

The collection areas were assembled into three categories: landfill sites (GTR and MER), roosts (GTR, MER, STK1, STK2, OR), and boneyards (GTR and PC). The landfill sites corresponded to the land areas where the waste materials were dumped on the ground. The roosting sites were actively occupied by Black Vultures during either the whole sample collection cycle (GTR and MER), or during the spring collection only (STK1, STK2, OR). The boneyards were large roosts where at least 50 birds were estimated to be deceased based on the number of carcasses, bones, and feathers detected at the site. Besides, birds from the large roost GTR were also known to be feeding at the landfill. A total of 107 samples were collected between May 2023 and March 2024 to obtain a representation of a full annual cycle. For each season, a total of 39 samples were collected, except in summer when the roosting site OR could not be sampled in time, and in winter when only the landfill sites were sampled. Noteworthy, due to inclement weather conditions, the winter sampling sites were completely flooded, and fecal samples could not be collected. More detailed information about GPS coordinates and collection date is available for each sampling area and sample type (Table S1).

### Sample collection

During each sampling period (i.e., season), three types of samples were collected: feces (22), background soil (72), and water (13) (Table S1). We sampled fresh fecal droppings from New World vultures, using sterile gloves and 50 mL centrifuge tubes. Vulture droppings were collected from vulture roosts, areas where these birds had been observed immediately before collection. Given the location and size of these droppings we are certain that the fecal droppings collected were those of Black Vulture.

At all sites, soil cores (0–10 cm) were collected in triplicate with a disinfected stainless steel sampling probe and transferred inside a small zip-lock bag. At each type of sampling location, controls were collected for soil samples (e.g., directly outside the waste management zone in MER) to verify whether the presence of bacterial communities is general to the environment or directly related to the presence of vultures. For each soil sample, moisture content was calculated from a 10 g field moist soil aliquot that was weighed, oven dried at 104 ◦C for 24 h and weighed again. Water (50 mL) was collected from runoff (PC) or horizontal runoff landfill water (GTR and MER) with a sterile pipette pump.

### Bacterial isolation and identification

Enterococci, thermotolerant *Escherichia coli,* and *Salmonella* spp. were assayed in all soil, water, and fecal samples. As previously recommended by [71, 74], 10-gram aliquots of soil and feces samples were suspended in either 90-ml sterile physiological saline (0.85% NaCl) or 90-ml tryptic soy broth (TSB) solutions and mixed for 30 s in a stomacher 400 Lab-blender (Seward). Similarly, water samples were processed by transferring 10 mL aliquots into 90 mL of either the saline or TSB solutions and mixed for 30 s using the stomacher. Following stomaching, serial dilutions (10^-1^, 10^-2^, 10^-3^) were immediately prepared, and the samples were transferred to their respective medium and incubated as previously described [71, 74].

*Escherichia coli* and enterococci bacterial isolations were performed using standard membrane filtration [72], while *Salmonella* spp. was assayed using a modified enrichment presence/absence [72] approach, and results were reported as present (detected) or absent (not detected), and colony forming unit (CFU) per 100 ml (water) or per gram (soil and feces). Briefly, *E. coli* was isolated through the use of the EPA 1603 method via modified mTEC agar, enterococci isolated via the use of EPA 1600 method via mEI agar, and *Salmonella* enriched via tryptic soy broth, Rappaport-Vasilidales R10 broth, semisolid Rappaport-Vasilidales agar, and final isolation to Hektoen enteric agar, all purchased via Neogen-Accumedia. For each presumptive isolate of enterococci, *E. coli*, and *Salmonella spp.*, one colony was selectively isolated, streak-purified, resuspended, and stored at -80◦C in 15% glycerol-TSB. The identity of each bacteria (*E. coli,* enterococci, and *Salmonella spp.*) was confirmed for all isolates through qPCR assays. Each glycerol-TSB isolate was incubated on TSA plates at 37°C overnight, and single colonies were transferred to individual tubes containing 100 µl dH_2_O using a toothpick. DNA was denatured in a heat block (100 °C) for 1 minute and centrifuged at 14,000 rpm for 1 minute. Sample isolates were assessed using the ABI PowerSybr PCR Mix in duplicate on an Applied Biosystems StepOnePlus real-time PCR system (Table S2). Information about primer sequences is also available as metadata (Table S3).

### Antibiotic resistance

Antimicrobial resistance of the enterococci, *E. coli*, and *Salmonella* isolates were assessed *via* the Kirby–Bauer disk diffusion method, using antibiogram criteria [71, 73]. Fresh colonies were obtained by streaking individual isolates onto 1 mL of tryptic soy agar (TSA) and grown at 35°C overnight. The isolates were then inoculated and incubated into TSB at 37 °C in a temperature-controlled incubator (150 rpm) for 2–3 hours. Following incubation, 100 mm Mueller Hinton Agar (MHA) plates (Neogen Corporation, Lansing, MI, USA.) were inoculated with each suspension using sterile cotton swabs. An automatic disk dispenser (BBL® Sensi-Disc® 8-place Dispenser, Becton, Dickinson and Company, Franklin Lakes, NJ, USA.) was used to dispense a total of 8 antibiotic susceptibility discs (Becton, Dickinson and Company, Franklin Lakes, NJ, U.S.A.) onto each MHA plate before being incubated at 37°C overnight.

Each enterococci isolate was tested for resistance to the following 12 antibiotics: erythromycin (E - 15 µg), neomycin (N - 30 µg), kanamycin (K - 30 µg), gentamicin (GM - 120 µg), cephalothin (CF - 30 µg), penicillin (P - 10 µg), ciprofloxacin (CIP - 5 µg), tetracycline (TE - 30 µg), vancomycin (VA – 30 µg), ampicillin (AM - 10 µg), clarithromycin (CLR – 15 µg), and amikacin (AN - 30 µg). For both *E. coli* and *Salmonella*, resistance to the following 12 antibiotics was tested: imipenem (IPM - 10 µg), neomycin (N - 30 µg), kanamycin (K - 30 µg), gentamicin (GM - 120 µg), cephalothin (CF - 30 µg), levofloxacin (LVX – 5 µg), ), ciprofloxacin (CIP - 5 µg), tetracycline (TE - 30 µg), cefoperazone (CFP - 75 µg), ampicillin (AM - 10 µg), polymyxin-B (PB – 300 µg), and amikacin (AN - 30 µg). These different classes of antibiotics, to wit, β-lactam (P, AM, IMP), cephalosporin (CF, CFP), glycopeptide (VA), peptide (PB), macrolide (E, CLR), aminoglycoside (AN, GM, N, K), tetracycline (TE), and quinolone (CIP, LVX), were chosen for their wide use in agriculture and patient care, and consequently, represent the majority of antimicrobial classes [27, 40, 44].

Three control reference strains, *Pseudomonas aeruginosa* (ATCC 27853), *Staphylococcus aureus* (ATCC 25923), and *E. coli* (ATCC 25922), were used at each antibiotic disc diffusion session, to ensure consistency of the process. For each MH tested plates, images were captured using a UVP GelDoc-It imaging system (Analytik Gena US LLC, Upland, CA, USA), and the resistance (or susceptibility) to each antibiotic was determined by measuring the diameters (mm) of the inhibition zones, using the software ImageJ. Following the Clinical and Laboratory Standards Institute (CLSI) standards, the susceptibility of bacteria to each antimicrobial agent was interpreted as Susceptible (S), Intermediate (I), and Resistant (R).

### ARGs analysis

DNA was extracted from the soil and fecal samples using the FastDNA spin kit for Soil (MP Biomedicals, Solon, OH, U.S.A.), and the FastDNA spin kit for feces (MP Biomedicals, Solon, OH, U.S.A.). While some modifications in the DNA extraction protocol (e.g., binding, washing, and elution steps) were made from the manufacturer’s suggestions (Table S2), the recommended Fastprep machine (Fastprep24, MP BIO) was used for the bead-beating step. PCR-ready genomic DNA products were eluted in either 60 µl (feces) or 100 µl (soil) DES/TES solutions, and DNA concentrations were measured with the QuBit dsDNA HS Assay kit (Invitrogen, Life Technologies, U.S.A.) through fluorometry (Qubit, Life Technologies, U.S.A.). Each DNA isolate was diluted by 10 or 100-fold. When concentrations were too low to be measured, ½ dilutions were also made. All samples were then stored at -20°C until qPCR testing.

Based on the antimicrobial resistance phenotypes observed in our study, we analyzed the presence of the following 6 ARGs (Table S3): tetracyclines (*tet*A and *tet*B), erythromycins (*erm*A and *erm*F), β-lactam (*bla*TEM), and sulfonamide (*sul*1). Resistance to the class 1 integron gene (*int*1I) and the 16S ribosomal RNA (16S) was also measured. For each sample, DNA dilutions of 10^-1^ (or 1⁄2 for low concentration samples) were used to test for resistance to *tet*A, *tet*B, *erm*A, *erm*F, *bla*TEM, *sul*1, and *int*1I. To account for the expected high gene copy number of 16S rRNA in our samples, resistance to 16S was assessed using DNA dilutions of 10^-2^ (or 10^-1^ for low concentration samples). Each sample was tested in duplicate using the ABI PowerSybr PCR Mix on an Applied Biosystems StepOnePlus real-time PCR system (Table S2).

### Statistical analyses

All the statistical analyses were performed in RStudio (version 4.3.2). For each statistical test, a list of *a priori* models was made, and the best models with strongest statistical support were selected using the R-squared values generated from the Akaike Information Criterion (AIC). Prior statistical analyses, normality tests (e.g., Shapiro-Wilk Normality Test) were performed on our datasets, using the R packages *ggpubr* (version 0.6.0) and *stats* (version 3.6.2). Moisture contents of soil samples were compared across seasons and between types of sampling sites, using the non-parametric Kruskall-Wallis test, and the pairwise post hoc multiple comparisons Dunn test, using the R package *rstatix* (version 0.7.2). For both enterococci and *E. coli*, bacterial count (CFU) was standardized by the dilution factors (e.g., count*10 for dilution 10^-1^), and transformed into log(x+1). The non-parametric Kendall rank correlation coefficient was used to estimate the rank-based measure of association between the CFU of *E. coli* and enterococci*.* In contrast, the isolation of *Salmonella* was reported through presence (1) or absence (0). For each isolate, bacterial abundance was compared across seasons, sampling sites, and sample types (soil, feces, and water). All tests were considered significant at α ≤ 0.05.

For each isolate of *Enterococci, E. coli*, and *Salmonella*, resistance profiles to the 12 chosen antibiotics were also compared across the season, type of sample, and sampling locations, using a Kruskall-Wallis test coupled with pairwise post hoc Dunn tests. The susceptibility of bacteria to each antimicrobial agent was interpreted as Susceptible (S), Intermediate (I), and Resistant (R). Heatmaps and dendrograms were plotted to represent the hierarchical cluster analysis using the R packages *heatmaply* (version 1.5.0) and *gplots* (version 3.1.3.1). To analyze multidrug resistance (MDR), binomial resistance values of each isolate were determined and grouped into five categories of resistance to ≥1, ≥2, ≥3, ≥4 and ≥5 antibiotic categories. MDR profiles across seasons, sample types, and sampling locations were visualized as a Circos diagram using the R package *circlize* (version 0.4.16). A hierarchical cluster analysis was also made to assess the level of similarity of MDR profiles across antimicrobial classes using the R packages *ggplot* (version 3.4.4) and *ggdendroplot* (version 0.1.0). The main association between antimicrobial classes was shown in a heatmap associated with a dendrogram based on the Pearson correlation coefficient.

Differences in the abundance of antimicrobial resistance genes across samples were determined with the Bray-Curtis similarity index *via* a Nonmetric multidimensional scaling (NMDS) and a one-way analysis of similarity (ANOSIM), using the R package *vegan* (version 2.6.4). The degree of discrimination among samples was quantified by reporting the generated R statistic, which ranges from -1 to 1, for similarities within and across the various groups tested (i.e., seasons, sample types, and sampling locations). An *R* value close to 1 means high dissimilarities within groups, while R = -1 highlights high dissimilarities among each tested group. In contrast, when the *R* value is approximately zero, similarities within groups are similar to differences between groups. In parallel, we also ran an indicator species analysis (ISA), using the R package *indicspecies* (version 1.7.14), to identify which ARGs were more associated with the different variables tested in the NMDS.

## Conclusions

The present study supports the hypothesis that in wildlife antimicrobial resistant bacteria and pathogens can be excreted in feces and persist in the environment. As such, we agree that New World vultures can be considered suitable sentinels for AMR and MDR surveillance. Moreover, we confirm that human-altered habitats, such as landfills, may be reservoirs for the occurrence and spread of resistant *E. coli,* enterococci*,* and *Salmonella*. Yet, we also show that “natural” sites can pose a similar risk of AMR/MDR as landfills. Even though a prudent management of anthropogenic wastes to prevent wild animals accessing human wastes should remain a key strategy, our results challenge the assumption that solely reducing anthropogenic pollution will be sufficient to prevent the transmission of AMR to wildlife. Stepping outside of the ‘blame game’ of landfills, we want to emphasize that further targeted research is needed to quantify and compare resistance levels in natural *versus* urban environments. Because our results coincide with the upward trend of AMR/MDR detection worldwide, we highlight that further research is needed to better understand the dynamics and risks associated with environmental AMR. For instance, looking at sex and age differences, comparing microbiomes between healthy and diseased birds, and investigating the role of animal dispersal and seasonal complexities, are worthy of future consideration. While we acknowledge several limitations in our approach (e.g., uneven number of samples across seasons and heterogeneity in the sample types), our work paves the way for the development of future risk assessment and mitigation strategies. The addition of information about food sources or the presence of supplementary feeding stations, as well as movement patterns of Neo World vultures, should be considered to develop further collaborative and multidisciplinary research under the One Health paradigm.

## Supplementary Information

Supplementary Material 1.

Supplementary Material 2.

Supplementary Material 3.

## Data Availability

The original contributions presented in the study are included in the article/supplementary materials.
